# Graph-Based Inter-Subject Pattern Analysis of fMRI Data

**DOI:** 10.1371/journal.pone.0104586

**Published:** 2014-08-15

**Authors:** Sylvain Takerkart, Guillaume Auzias, Bertrand Thirion, Liva Ralaivola

**Affiliations:** 1 Institut de Neurosciences de la Timone UMR 7289, Aix Marseille Université, CNRS, Marseille, France; 2 Laboratoire d'Informatique Fondamentale UMR 7279, Aix Marseille Université, CNRS, Marseille, France; 3 Parietal Team, INRIA Saclay - Ile-de-France, Saclay, France; Universiteit Gent, Belgium

## Abstract

In brain imaging, solving learning problems in multi-subjects settings is difficult because of the differences that exist across individuals. Here we introduce a novel classification framework based on group-invariant graphical representations, allowing to overcome the inter-subject variability present in functional magnetic resonance imaging (fMRI) data and to perform multivariate pattern analysis across subjects. Our contribution is twofold: first, we propose an unsupervised representation learning scheme that encodes all relevant characteristics of distributed fMRI patterns into attributed graphs; second, we introduce a custom-designed graph kernel that exploits all these characteristics and makes it possible to perform supervised learning (here, classification) directly in graph space. The well-foundedness of our technique and the robustness of the performance to the parameter setting are demonstrated through inter-subject classification experiments conducted on both artificial data and a real fMRI experiment aimed at characterizing local cortical representations. Our results show that our framework produces accurate inter-subject predictions and that it outperforms a wide range of state-of-the-art vector- and parcel-based classification methods. Moreover, the genericity of our method makes it is easily adaptable to a wide range of potential applications. The dataset used in this study and an implementation of our framework are available at http://dx.doi.org/10.6084/m9.figshare.1086317.

## Introduction

Functional Magnetic Resonance Imaging (fMRI) is a modality that has proved extremely useful for understanding brain function as it offers the possibility to map cognitive processes to brain activation patterns. Traditional univariate analysis methods of fMRI data process each voxel separately to perform *forward inference* [1], that is, identify those voxels that show an activation profile significantly associated with a given task. With the recently proposed application of multivariate pattern recognition methods to fMRI data, one can also make *reverse inference*, that is, predict a behavioral variable directly from the imaging data, as in the pioneering work described in [2]. This new approach, often referred to as *multi-voxel pattern analysis* (MVPA), *brain decoding* or *mind reading*, has received an increasing amount of attention over the last few years. The vast majority of papers published so far (see reviews [3]–[5]) study the organization of cortical representations, a problem particularly suited for MVPA since such representations arise from neural activity distributed across networks that can cover a large number of fMRI voxels. Another problem that can be addressed through MVPA techniques is to examine the consistency of patterns across tasks by testing whether patterns observed in a given task may arise in different tasks, as in [6], [7]. Finally, one can also use MVPA to characterize patient groups from fMRI data, in order to identify putative fMRI biomarkers that could be used in diagnosis tools [8]–[10]. All these applications ask for constructing *group-invariant characterizations*. Most studies published until today address this question with a two-level inference, performing MVPA within subject, and testing the consistency of within-subject classification scores across individuals. However, this limits the interpretability of the results because within-subject MVPA often relies on sub-voxel idiosyncratic information [11]. It is therefore of the highest interest to address this question more directly by performing inter-subject MVPA, i.e. by looking for features that are common across subjects and learning a decision rule on data recorded in a set of subjects to use it on data from different subjects.

### Challenge

The potentially large inter-individual variability represents a major challenge to construct group-invariant representations that will allow for successfull inter-subject MVPA. Only few studies have directly addressed this question. Most rely on full brain analysis, using large-scale features that are stable across subjects after spatial normalization [1]. While a recent study proposes to use a multi-task framework to handle large scale inter-subject variability [12], all the others focus on the feature construction/selection: several papers use univariate feature selection with different criteria (relative entropy in [13], most active or discriminative voxels in [14] and [15]); others summarize the signal present in a set of regions by their mean, using, e.g., cubic regions [16], anatomically defined regions [17] and [18], or functionally defined parcels [17]; [19] uses principal component analysis; finally [20] and [21] use sparse learning methods that automatically select features. When examining patterns at a finer spatial scale, inter-individual variability is yet larger and performing such inter-subject predictions becomes even more challenging. At such scale, the alignement between cortical folding and the underlying functional organization vary between subjects [22], [23], in a way that the potentially poor voxel-to-voxel correspondance provided by spatial normalization procedures limits the generalization power of classifiers that use voxel values as features [24]. To our knowledge, only two studies describe methods specifically designed for inter-subject classification without the need for spatial normalization. The first one [25] uses Procrustes transformations to maximally align, in a high-dimensional space, the spatio-temporal patterns recorded during a specific training experiment. The second one [26] is a discriminant analysis that projects the data (through a generalized PCA) onto multiple-subjects factorial maps designed to maximize class separation. Both these techniques do not enforce the preservation of the spatial organization of the input patterns to construct their latent space.

### Structured learning

In order to tackle the challenge posed by inter-subject variability, structural analysis schemes have proved efficient for forward inference group studies in neuroimaging, as described in [27], [28]. However, no such structural approach has been developed to perform reverse inference. Our goal here is to develop a learning framework that specifically aims at predicting a behavioral variable from imaging data while overcoming inter-subject variability by exploiting the structural properties of the input patterns. Such a framework should address three problems:

• What are the structures of interest? In neuroimaging, two main classes of elementary objects are used in such approaches: points (local maxima of activation [29]) or regions (clusters of activation [27], parcels [30]). These functional features can be represented into a graph to encode their relationships, as it is now classically done with connectivity-based models of functional or anatomical networks.

• How is the inter-individual variability conveyed? Regardless of the chosen feature type, models of inter-individual functional variability let their location [28] and intensity [31] vary across subjects.

• What learning method to use? Learning from structured data can be done with a wide variety of methods, among which, neural/deep belief networks [32], probabilistic/graphical models (such as Markov fields [27], hierarchical Dirichlet processes [31] or Conditional random fields [33]), or large margin kernel-based methods with appropriately engineered kernels (see [34], [35]).

### Contributions

**Figure 1:**
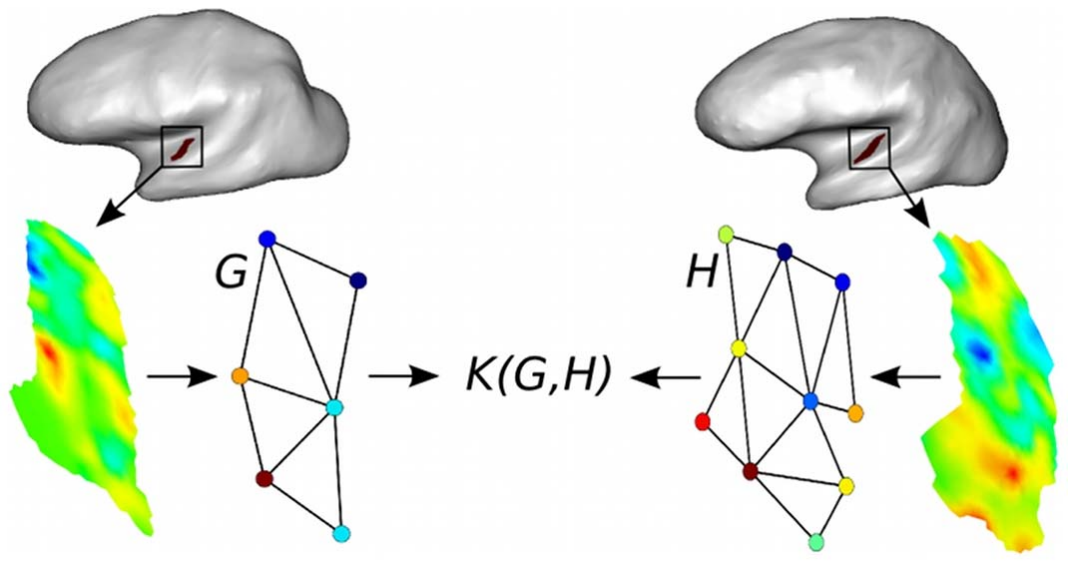
Our contributions: i) attributed graphs are learnt in an unsupervised manner to represent local functional patterns observed in unregistered subjects; ii) graphs similarities are evaluated by a custom-designed kernel, allowing to solve various problems (classification, regression, clustering).

In the present paper, we introduce a Graph-based Support Vector Classification (G-SVC) framework that respectively addresses the previous questions by i) using unsupervised learning to construct attributed graphs that represent fMRI patterns of activation; the nodes are patches of activation given by a parcellation algorithm; the graph edges carry the spatial relationships between nodes and their relevant characteristics (location and activation) are encoded as attributes of the graph nodes; ii) assuming that both attributes of the nodes can vary across subjects, i.e. that the inter-individual variability can be characterized along these two dimensions; iii) designing a graph similarity measure (a graph kernel) that is robust to inter-individual variability, and that makes it possible to perform supervised learning directly in graph space, for instance by using support vector classification. These contributions are summarized in Fig. 1.

While the use of graphical representations of fMRI data has seen a tremendous boost in the last decade with the fast development of connectivity analyses (see for instance [36], [37]), graph kernels have only recently been introduced in the neuroimaging field. The few studies that make use of graph kernels to solve neuroimaging learning problems address different sorts of questions (subject classification based on resting-state functional connectivity [38] or task-based fMRI [39], characterization of the mental state of the subject from its connectivity [40] or activation [41], [42] patterns), showing their potential versatility.

Our framework, for which a preliminary study was presented in [43], falls in the latter category. It is specifically aimed at overcoming the fine scale *functional variability* observed in a given region of interest for a task-based fMRI experiment, which is a key issue in understanding local neural representations [25], [44]. In such case where using spatial normalization is the bottleneck, our framework allows to explicitely take into account the different sources of inter-individual variability without requiring perfect cross-subject matching of brain anatomy, hereby alleviating the dependecy of the method to the registration accuracy. Furthermore, it can easily be tuned to address numerous problems provided one may have at hand a meaningful parcellation for the question of interest, as for instance in full brain resting state studies (see a review in [45]) or diffusion weighted-based segmentation of grey matter regions (as for instance in [46]).

## Materials and Methods

### 2.1 Graph-based Support Vector Classification (G-SVC)

**Figure 2:**
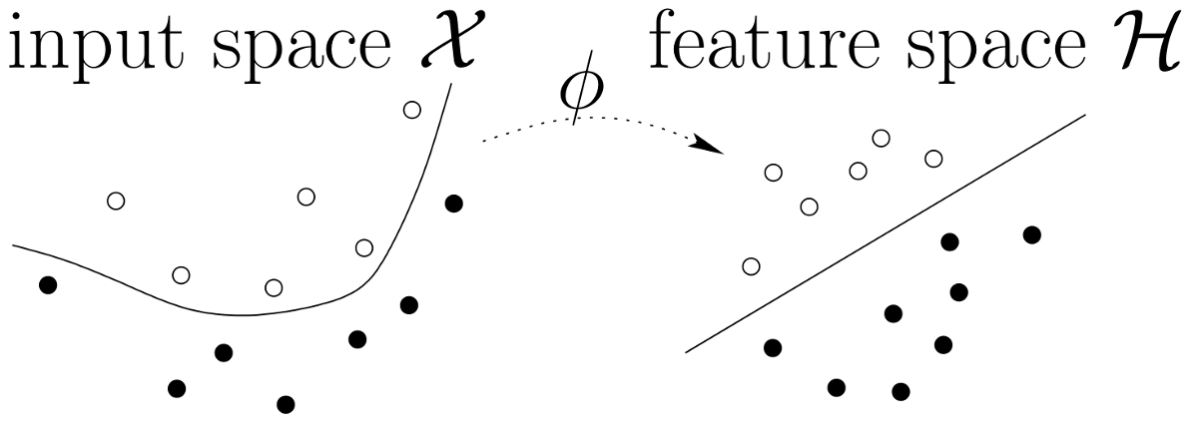
The kernel trick: the use of a positive kernel *K* implicitely maps data from some input space 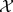 into a Hilbert space 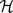 —thanks to the canonical mapping 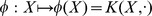
— where linear separation is possible.

The defining task that is usually addressed in inter-subject MVPA might be stated as the learning of a classifying function *f* able to reliably predict a categorical experimental variable *y* from fMRI data *X* recorded in a given set of subjects. In order to gain invariance with respect to the inter-subject variability and be able to generalize to data from new subjects, we use a graphical representation *X* of the input data (described below). Effective methods have recently emerged to learn from such structured data ([27], [31]–[35], and among those, similarity-based learning approaches (nearest-neighbors methods, kernel machines, relevance vector machines, …) have received much attention. We focus here on *support vector classifiers* [47], or *SVC*, because of their well-foundedness and their effectiveness in various application domains, including neuroimaging. Without entering into too much detail, the most prominent way to perform support vector classification works by solving the following quadratic problem (note that we here recall the binary approach to support vector classification; composition methods such as the well-known *one-vs-all* or *one-vs-one* strategies make it possible to directly build multiclass predictors from the binary method): 
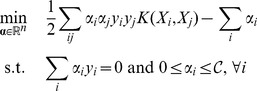
(1)the solution 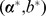 of which (where *b* ^*^ is given by the optimality conditions of the problem) defines a classifier *f* given by: 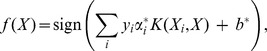
where 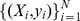 is the *training data* of labeled pairs (*X_i_*, *y_i_*), made of pattern *X_i_* and associated (binary) target *y_i_*, 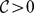 is a user-defined (soft-margin) parameter and *K* is a *positive kernel* function. This kernel function implicitly allows one to map the training patterns *X_i_*'s into a relevant Hilbert space (an idea, known as the ‘kernel trick’, that dates back to [48]) where large-margin linear classification is possible (see Fig. 2). Choosing/designing an appropriate kernel for the data at hand, is therefore the crux of using support vector classification for real-world applications, knowing that dealing with structured inputs merely requires the design of a sound kernel.

**Figure 3:**
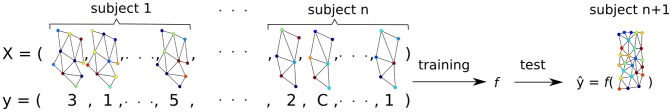
Inter-subject graph-based learning. Equipped with a graph construction scheme and a graph similarity function such as those designed in this paper, one can define numerous classifiers. The decision function *f* is learnt on a training set composed of labelled graphs (*X_i_*, *y_i_*), with 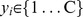 from a set of subjects, and can be used on graphs from another subject, potentially with a different number of nodes. We here use support vector machines to demonstrate the soundness of this approach when dealing with inter-subject variability of fMRI activation patterns.

In what follows, we describe how we build a graphical representation of functional patterns (section 2.2) and a graph kernel (section 2.3). With these tools, one can define numerous classifiers to perform inter-subject fMRI prediction (illustrated on Fig. 3); here, without loss of generality, we instantiate the support vector classification framework. Therefore, our graph construction scheme and graph kernel fully define our Graph-based Support Vector Classifier (G-SVC) framework.

We assume that for the considered task-based fMRI experiment, we have at our disposal for each subject: i) a pre-defined contiguous *region of interest* (ROI) 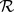, ii) the function 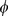 describing the BOLD activation at each experimental trial (each trial providing a different observation), and iii) a coordinate system 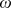 (in practice, we use a 2d cortex-based set of coordinates, which is more meaningful than working in the 3d volume [49]). Furthermore, we assume that the functional organization of 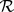 with respect to our experiment is consistent across subjects.

*Remark*. The way we use support vector classification in what follows departs a little bit from what is suggested by the theory that supports the use of SVM. Indeed, the classical framework assumes the training set 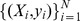 be made of identically and independently distributed random pairs, whereas the pairs we are going to work with may be dependent as different training pairs (*X_i_*, *y_i_*) could relate to the same subject. Carefully characterizing and taking into account these dependencies is an important challenge posed by many inter-subject prediction problem (see [50]) that goes beyond the scope of the present paper. Ideas taken from [51], [52], may lay the theoretical ground to build relevant and original approaches and constitute the main axis of our future researches. In any case, our use of SVM is frequently encountered in the literature, in e.g. information retrieval problems [53]–[55], where no particular care of such dependencies exist and still very good classification results are achieved.

### 2.2 Graphical representation of fMRI patterns

Here, we detail the unsupervised representation learning scheme that we use to derive graphical representations from fMRI activation patterns.

#### 2.2.1 Graphical representation - Parcellation of the ROI

Assuming that the ROI 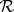 admits an underlying subdivision into a set of smaller and functionally meaningful sub-regions, the first step to construct our graphical representation consists in estimating a partition of 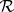 into a set of sub-regions or *parcels* [30]. Specifically, a parcellation 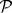 of 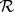, is a set 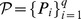 of *q* parcels so that the parcels verify: 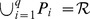 and 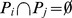 whenever 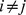.

We use Ward's hierarchical clustering algorithm to learn the parcellation in an unsupervised manner. The algorithm starts with one parcel at each point 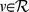, and iteratively merges two parcels into one so that the variance across all parcels is minimal, with the added constraint that two parcels can be merged only if they are spatially adjacent [56]. The input vector that we used is 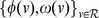; it combines the anatomical information provided by the coordinate system 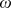, and the full functional information available for a given subject (i.e. the activation maps recorded at each trial for all experimental conditions). Incorporating the anatomical information on top of the functional features acts as a spatial regularization process in the search for functionally meaningful units, which makes the parcellation more robust to the low contrast-to-noise ratio of the functional data usually encountered when using MVPA. The resulting parcels constitute the elementary functional features that are the base elements of our approach.

#### 2.2.2 Graphical representation - Graph nodes and edges

We use 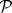 as the set of nodes of our graphical representation, i.e. each parcel defines an elementary functional feature of the pattern that is represented by a node of the graph. The set of edges is represented by a binary adjacency matrix 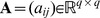, where *a_ij_* = 1 if parcels *P_i_* and *P_j_* are connected, and *a_ij_* = 0 otherwise. In this work, since we assume that the topological properties of the patterns are stable across subjects, we use spatial adjacency as the criterion to decide whether two nodes are connected (i.e. *a_ij_* = 1 if 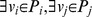 so that *v_i_* and *v_j_* are neighbors). This defines a region adjacency graph [57] where the structure of the graph encodes the spatial organization of the parcels. Note that our method is also fully valid if one had used other criteria (for instance functional connectivity) to define the edges of the graph.

#### 2.2.3 Graphical representation - Activation attributes

In a parcel *P_i_* and for a given observation (i.e. experimental trial), the activation values 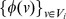 are summarized by their mean inside the parcel, that we note 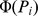. We note 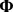 the vector 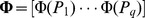 of activation attributes. Note that, more generally, we may summarize the activation values measured in *P_i_* using a feature vector 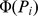; this vector could include the mean, the variance, the skewness, …, or any other summary statistics.

#### 2.2.4 Graphical representation - Geometric attributes

The geometric information of parcel *P_i_* is summarized by a feature vector 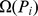, computed from the locations 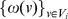. In this study, we use the coordinates of the center of mass of the ROI, computed within the coordinate system 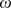. We note 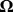 the matrix of geometric features 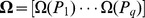. As before, richer geometric information, accounting for instance for the shape of the parcel, may be considered.

#### 2.2.5 Graphical representation - Full graphical model

**Figure 4:**
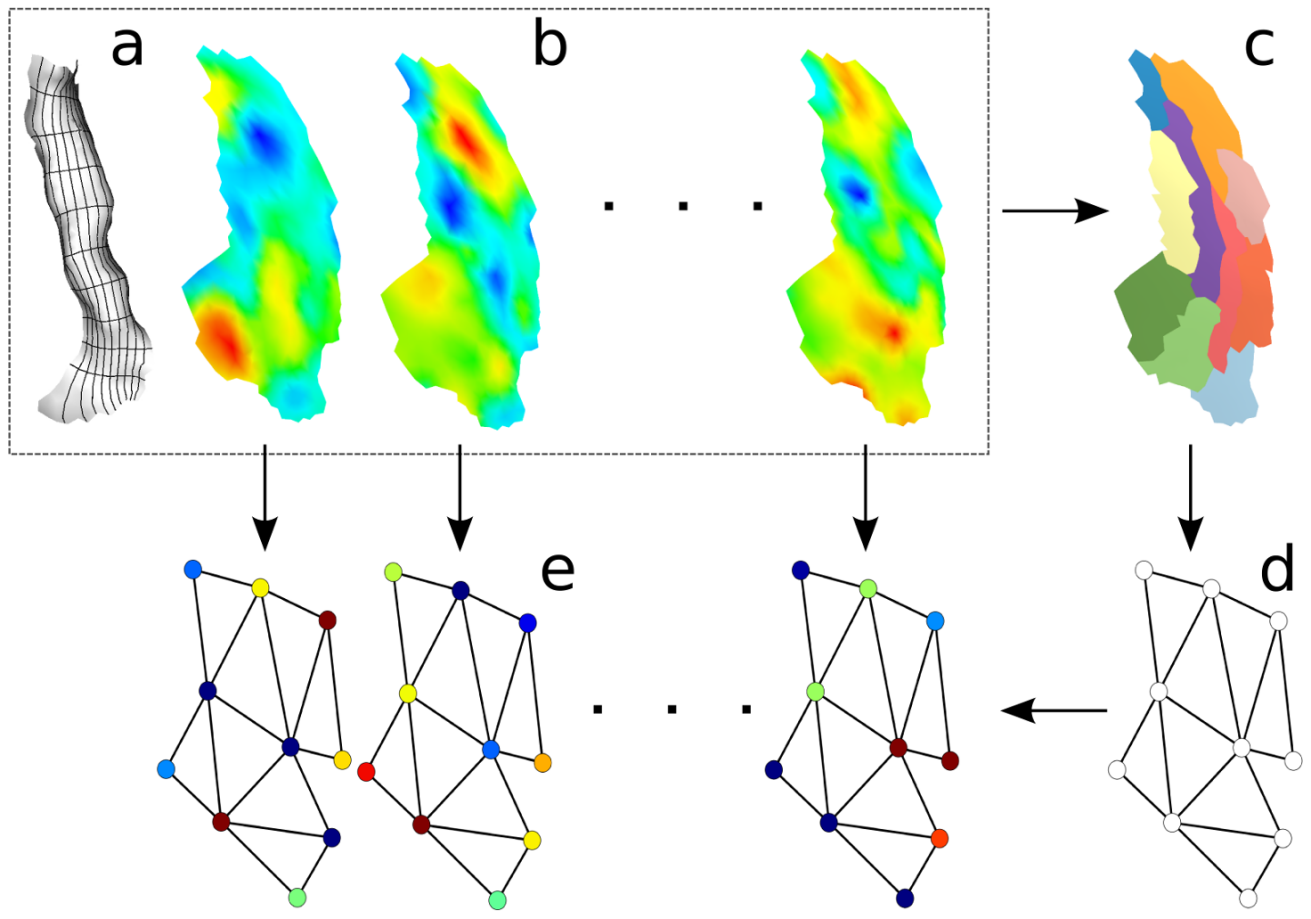
Construction of our graphical representation of functional patterns. Starting from a local coordinate system 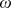 (illustrated in **a** as a grid on the local cortical mesh) and the functional activation maps 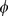 (displayed in **b** as overlays on the flattened mesh), we produce a parcellation 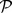 (shown in **c**) which gives the graph structure **d** with the location of the nodes. The activation values of each functional example are then mapped onto the nodes to produce the attributed relational graphs shown in **e**, which carry all the necessary information through its structural, geometric and activation features.

Using these definitions, we have defined an attributed relational graph [58] 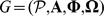 and described how to learn such graphical representations in an unsupervised manner. These graphs fully represent the functional patterns recorded within the ROI 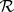 and carry activation, geometric and structural information, as illustrated in Fig. 4.

### 2.3 Graph similarity

Among the various frameworks that exist to learn from structured data, what makes similarity-based methods popular is that the difficulty of the learning process is transferred to that of defining a similarity function on the space of structured objects at hand. It turns out that a plethora of graph similarity functions exist, defined with respect to the number of edit operations needed to transform one graph to another [59], the number of common subgraphs of certain type (walks [60], trees [61], graphlets [62]), or the similarity between vector representations of graphs (see for instance [63]).

Here, we decide to use a positive kernel as a similarity measure between graphs: this makes it possible to envision the use of kernel-based machine learning algorithms (such as the *support vector classifiers* described above), which have proved efficient in this context [5] and offer solid theoretical guarantees. When choosing or designing a graph kernel for a given application, one need to find a good compromise between expressivity (i.e. the ability of the kernel to capture the features of interest in the available graphs) and computational efficiency [64]. The recently developed Weisfeiler-Lehman graph kernel [65] offers such properties (which has made it the kernel of choice for several recent neuroimaging applications [39], [41], [42]) but its applicability is limited to labeled graphs. Since the key features of our graphical representations are carried by the *real-valued* attributes of the nodes, we want to avoid having to quantify the values of these attributes into discrete labels, which would imply loosing both some precision and the also the structure provided by real-valued attributes. We therefore decided to construct a dedicated kernel. In order for our kernel to provide a good balance between the two aforementioned criterion, we followed two directions: on the one hand, our design builds upon the work of [60] which layed the ground for the construction of efficient walk-based kernels computable in polynomial time; on the other hand, the expressivity of our kernel is based on an intuitive design scheme which aims at exploiting each type of graph features available in our representation, and in particular its real-valued node attributes. Below, we describe our design step by step as an instantiation of the generic family of *R*-convolution kernels [66], which are defined as: 
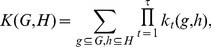
(2)where *G* and *H* are two graphs, 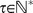 is the number of base kernels *k_t_*, which act on subgraphs *g* and *h* (for simplicity reasons, we here use walks of length one; note that the definitions below are directly extendable to other types of subgraphs).

For two graphs 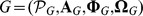 and 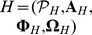, we note *g_ij_* and *h_kl_* two pairs of nodes (i.e. walks of length one) in *G* and *H*, respectively; let *q_G_* and *q_H_* be the number of nodes in *G* and *H*, respectively – note that *q_G_* and *q_H_* may be different. Given the nature of our graphical representation, we define 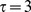 elementary kernels *k_s_*, *k_g_* and *k_a_*, respectively acting on structural, geometric and activation information, and thus covering all characteristics of the graphs.

#### 2.3.1 Graph similarity - Structural kernel

Because the structure of our graphical representations encodes the spatial adjacency of the parcels and because we assume that the spatial organization of the functional patterns is consistent across subjects, we include a first base kernel *k_s_* which aims at valuing the structural similarity of *G* and *H*. We simply adopt the linear kernel on binary entries 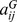 and 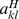 of the adjacency matrices **A** *_G_* and **A** *_H_*: 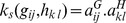
(3)


It gives 1 if 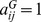 and 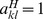, and 0 otherwise, meaning that the other base kernels are only taken into account if *g_ij_* and *h_kl_* are both actual edges. Our kernel in fact compares each edge of a graph to all edges of the other graph.

#### 2.3.2 Graph similarity - Geometric kernel

Kernel *k_g_* acts on the geometric attributes, i.e. the location of the graph nodes within the coordinate system 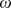. The goal of this kernel is to match edges across graphs. To allow for inter-individual differences, we implement a soft matching by using the following product of Gaussian kernels: 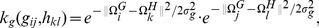
(4)where 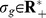, and 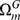 (resp. 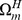) is the *m*th column of 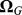 (resp. 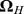). The contribution of the following base kernel is therefore be weighted by this soft matching term, and quasi-zero if the considered edges are not close to each other.

#### 2.3.3 Graph similarity - Activation kernel

Finally, base kernel *k_a_* is the heart of the functional pattern comparisons since it handles the functional activation information which carries the discrimative power for our classification task. This kernel measures the similarity of the activation levels recorded in parcels of *g_ij_* and *h_kl_*. As with *k_g_*, we use a product of Gaussian kernels: 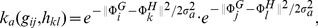
(5)where 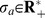 and 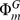 (resp. 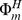) is the *m*th column of 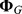 (resp. 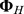). Using such kernel allows for variations in the activation attributes across subjects.

#### 2.3.4 Graph similarity - Resulting kernel

With the definitions of *k_s_*, *k_g_* and *k_a_*, we may define the resulting kernel: 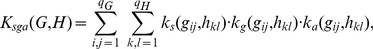
(6)


This kernel includes two parameters 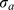 and 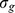, that are the bandwidths of the activation and geometrical base kernels. In standard learning problems working with vectorial inputs, a classical heuristic to estimate the value of the bandwidth of a Gaussian kernel consists in choosing the median euclidean distance between all observations in the training dataset [67]. Here, we use this heuristic by choosing the median euclidean distance between activation and geometric (respectively) attributes of all nodes and all observations (i.e. all graphs) in the training set.

### 2.4 Datasets

#### 2.4.1 Datasets - Artificial data

The generative model described here creates artificial datasets that allows to precisely evaluate G-SVC. Also not designed to simulate patterns with a spatial organization as complex as in real data, the important point here is that these patterns contain variations across subjects with respect to two dimensions, the location of functional features and their activation levels, which makes these artificial datasets realistic for that matter and challenging for inter-subject learning algorithms. By parametrically choosing the amounts of variability along these two dimensions, we are able to study the robustness of G-SVC to such functional variability.

**Figure 5:**
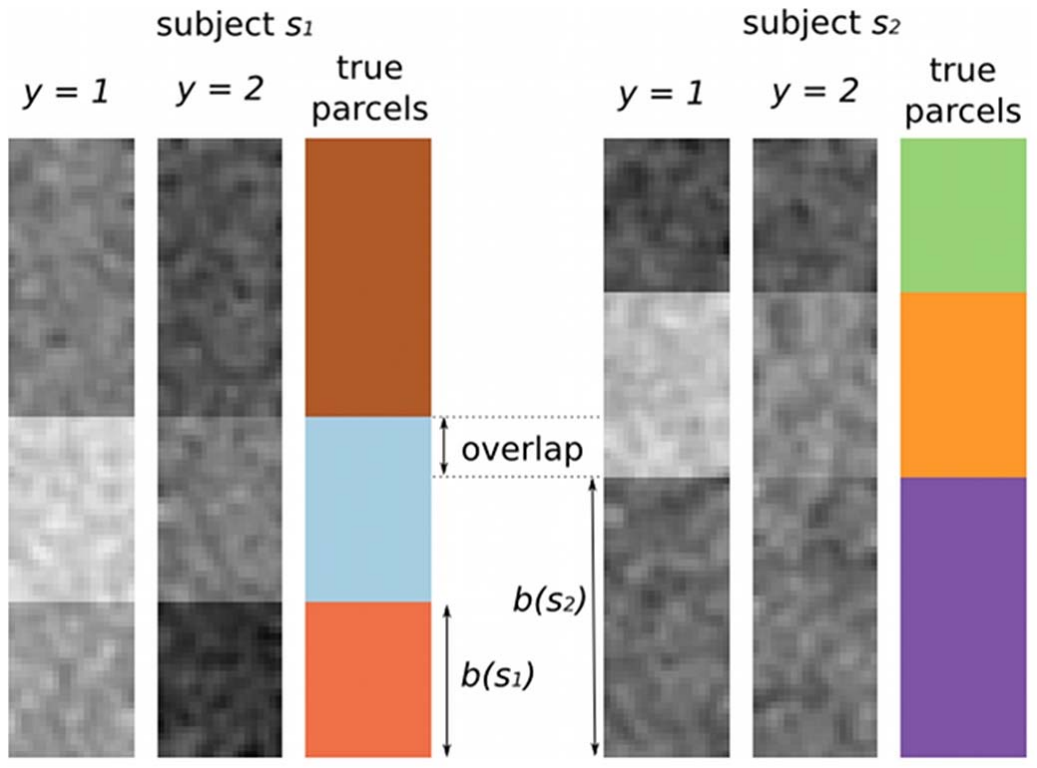
Artificial datasets: example patterns, with the true underlying parcellations used to generate the data. The inter-individual variability is controlled by two parameters: i) the locations of the middle parcels, which induces their overlap, here 33% (variability in geometric attributes); and ii) the value of 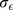, which induces the amount of variation in the mean intensities observed in the parcels (variability in activation attributes). Although the parcels are matched across subjects by construction, the colors of the true parcels are chosen different in the two subjects to illustrate that G-SVC does not need an a priori match.

As illustrated on Fig. 5, the artificial patterns are created on a rectangular support (20×100 pixels) corresponding to a given ROI 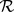 in the brain. We therefore use the trivial coordinate system 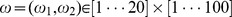. For a subject *s*, we directly simulate an observation *i* of the function 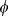 as 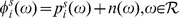. The *pixel noise* 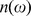 is added to make the patterns more realistic; it is generated by drawing values from a normal distribution 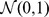, which are then smoothed by a 2D Gaussian filter, with FWHM of 2.35 pixels. The *true underlying pattern* 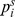 is a piecewise constant function given by 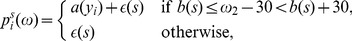
(7)


These patterns are therefore composed of three rectangular parcels in a vertical layout and cover the full region. The top and bottom parcels are *not active* (activation level close to zero), while the middle one is *active*; for trial *i*, its level of activation *a*(*y_i_*) depends on the experimental condition *y_i_*; two conditions were simulated, with *a*(*y_i_*) = 1 or 2 respectively, producing two classes of patterns. The variability between the two simulated subjects *s*_1_ and *s*_2_ is introduced by i) changing the position of the middle parcel: we used *b*(*s*_1_) = 20 in all cases and 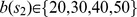, resulting in different amounts of overlap (100%, 67%, 33%, 0%) of this parcel across subjects; and ii) adding a Gaussian random variable 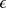 with distribution 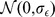 to the activation levels of each parcel, using 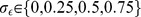. This respectively induced variability in the location and intensity of the discriminant functional characteristics of these patterns, i.e. the middle pattern. With four levels for each of these two types of variability, we obtain sixteen quantified cases, hereafter called *variability cases*; for each of these, we generate twenty datasets (each corresponding to a random draw of the values of 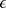 in each parcel and each condition, for each subject) comprising fourty trials (ten trials per condition per subject, each trial being the result of a different realization of the pixel noise 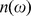 on the full pattern).

#### 2.4.2 Datasets - Real data

In order to test our framework on real data, we need an fMRI dataset for which it is known that functional patterns exhibit strong inter-individual differences at fine spatial scale but present a consistent functional organization across subjects. With that goal in mind we here study data recorded in an experiment designed to study the functional organization of the human auditory cortex. The experimental protocol was approved by the local ethics committee of Aix Marseille Universit (CCPPRB 01035), and written informed consent was obtained from all participants before the experiment. While the tonotopic property of the auditory cortex should result in a reproducible topographical organization of the activation maps across subjects, it has been shown that these functional maps suffer from a large variability across subjects [68], [69]. Such dataset therefore represents an adequate challenge for our framework.

For each of the ten subjects, a T1 image was acquired (1 mm isotropic voxels). Each stimulus consisted of a 8 s sequence of 60 isochronous tones covering a narrow bandwidth around a central frequency 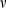. There were five types of sequences (i.e. five conditions 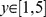 corresponding to five classes of patterns), each one centered around a different frequency 
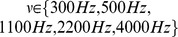
, with no overlap between the bandwidths covered by any two types of stimuli. Five functional sessions were acquired, each containing six sequences per condition presented in a pseudo-random order. Echo-planar images (EPI) were acquired with slices parallel to the sylvian fissure (repetition time = 2.4 s, voxel size = 2×2×3 mm, matrix size 128×128).

The preprocessing of the functional data, carried on in *SPM8* [1], consisted of slice timing correction and realignment. Then, a generalized linear model was performed (using *nipy* [70]) with one regressor of interest per stimulus. The weights of these regressors (beta coefficients) were used to compute the inputs of the classifiers because they provide a robust estimate of the response size for each stimulus [71]. We then used *freesurfer* [72] to extract the cortical surface from the T1 image and automatically delineate the primary auditory cortex (Heschl's gyrus) as it is defined in the Destrieux atlas [73], thus obtaining two cortical ROIs 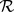 for each subject (one in each hemisphere). Note that this definition of 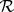, which implictly uses a spatial normalization, was chosen because it is fully automatic; other strategies working in the subjects' native spaces (manual drawing on the anatomy, functional definition) could also have been used.

In order to compute the graphical representations and apply our G-SVC framework, one need to define the function 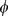 and a coordinate system 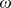. For this, we fully work in the subject's native space, i.e. without having to perform spatial normalization of the data into a common space. The function 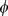 is the result of two operations executed in *freesurfer*: first, the beta maps obtained above are projected onto the subject's individual cortical mesh, and second, a slight spatial smoothing is performed along the cortical surface (equivalent FWHM of 3 mm). The values of 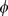 are then linearly normalized to the [0,1] interval within the ROI 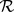. Several examples (for different observations) of the values of 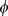 within the region 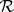 are shown on Fig. 4. Furthermore, since Heschl's gyrus has a rectangular-like shape (with another region of the Destrieux atlas on each side), we can define a 2D local coordinates system through a conformal mapping of 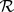 onto a rectangle (with a local version of the method described in [74]), defining 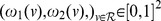. It is illustrated as a coordinate grid on Fig. 4. Note that forcing 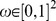 separately for each subject allows dealing with the case where the size and shape of 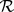 is different across subjects.

### 2.5 Evaluation framework

In this section, we briefly describe the experiments that we perform, the state of the art methods chosen to benchmark our G-SVC framework, as well as the methodology used to compare the performances of the different algorithms.

#### 2.5.1 Evaluation – Experiments

The first set of experiments consists in testing G-SVC and state-of-the-art vector-based methods on the artificial datasets. Since in these datasets, the amount of inter-individual variability is parametrically controlled along two dimensions (the location and activation levels of functional features in the pattern), studying the differential performances of G-SVC and benchmark methods for each *variability case* allows identifying the type(s) and amount(s) of functional variability for which G-SVC offers improved robustness. Then a series of experiments conducted on the real fMRI dataset makes it possible to i) overall compare the performances of G-SVC to those produced by state-of-the-art vector-based methods; ii) evaluate the influence of the number of graph nodes, compare the performances of G-SVC to those produced by standard parcel-based methods and examine the influence of working with individual vs. group parcellations; iii) test the robustness of G-SVC when its inputs are graphs with different numbers of nodes; iv) examine the usefulness of each of the three base kernels; and v) assess the stability in the estimation of the values of the kernel hyper-parameters.

#### 2.5.2 Evaluation - State-of-the-art vector- and parcel-based methods

In order to benchmark our G-SVC framework, we compare its performances to state-of-the-art inter-subject classification methods. The standard strategy to solve such inter-subject problem is to obtain a point-to-point mapping across individuals for the spatial domain of interest. This indeed allows flattening a pattern into a feature vector that is used as input for the classification algorithms. In our artificial datasets, the rectangular regions are matched across subjects by construction. In the real experiment where the regions might be slightly different from subject to subject, such feature vector can be constructed using a spatial normalization procedure, i.e. by bringing the data from all subjects into a common standard space. We here used the surface-based normalization process available in *freesurfer*, which provides a vertex-to-vertex correspondance across all subjects in the common *fsaverage* space. The function 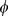 that was defined in each individual subject's space (see section 2.4) is resampled into this common space, and its values within the ROI (defined as the intersection of all the individual regions projected in the common space) makes up the common feature vector. Several classification algorithms, chosen because they have shown to be efficient for MVPA, are then tested, each time with a large set of values for their respective hyper-parameters: *1)* linear SVC; *2)* nonlinear SVC, with Gaussian (with 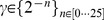) and polynomial (of order 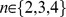) kernels; *3) k*-nearest neigbors (with 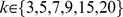); *4)* logistic regression with *l*_1_ and *l*_2_ regularization (with weight 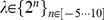). The parameter sets were empirically selected to ensure capturing the optimal performances of each of these algorithms for all the experiments described above.

Moreover, we also tested parcel-based methods as benchmarks for G-SVC. Once the data is projected in the standard space described above, one can compute a group parcellation for all subjects by using the anatomo-functional parcellation algorithm described previously, but with functional input features coming from all available subjects at training time. The parcels are thus naturally mapped to one another across subjects, and we can use a feature vector composed of the mean activation within each parcel (equivalent to the graph activation attributes 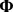) as input to any of the classification methods described above. Furthermore, using this group-parcellation, one can also construct graphical representations and use our kernel to perform inter-subject predictions; we denote this method as G-SVC^g^, as opposed to G-SVC when using individual parcellations. Comparing G-SVC with the results obtained with G-SVC^g^ and the other parcel-based benchmark methods was sued to clarify the role of using graphical representations learnt from individual parcellations and the usefulness of our graph metric itself.

#### 2.5.3 Evaluation - Performance evaluation and algorithms comparison

Amongst the wide range of metrics available for measuring the performances of classification methods, we selected the global classification accuracy, i.e. the fraction of correct predictions amongst all attempted predictions (one reflecting a perfect prediction score). Indeed, the design of both the artificial and real datasets used in this study yield balanced classes (identical number of observations in each class and each subject), and it has been shown that the global classification accuracy is perfectly adapted to such case [75]. For G-SVC, we report the global classification accuracy for different values of its hyper-parameters; for the benchmark methods, we report the highest accuracy obtained across all values of their hyper-parameters, thus putting G-SVC in the hardest possible case for performance comparison.

Since the different observations recorded in a given subject can be correlated, it is crucial to use a testing dataset composed of observations from subjects that were not part of the training dataset. We perform a leave-one-subject-out cross-validation and look at the average classification accuracy across folds, which is a natural strategy to measure the performance of an inter-subject classification algorithm. Assessing the significance of such an average classification accuracy and comparing different methods using the same scheme require a carefully elaborated evaluation method, that should for instance avoid employing Student's *t test* [76]. A solution is to use non-parametric tests. Here, we focus on comparing algorithms, and we apply two permutation tests that allow estimating the distribution of the null hypothesis that the algorithms perform identically. The first test (hereafter called *test1*), described in [77], allows to compare the performance scores of two algorithms by generating random sign permutations of the paired performance differences. The second one (hereafter called *test2*), described in [78], is a randomized ANOVA that allows to compare curves describing the performance of several algorithms in function of the value of one hyper-parameters shared by the different methods.

## Results

### 3.1 Results on artificial data - G-SVC vs. vector-based methods

**Figure 6:**
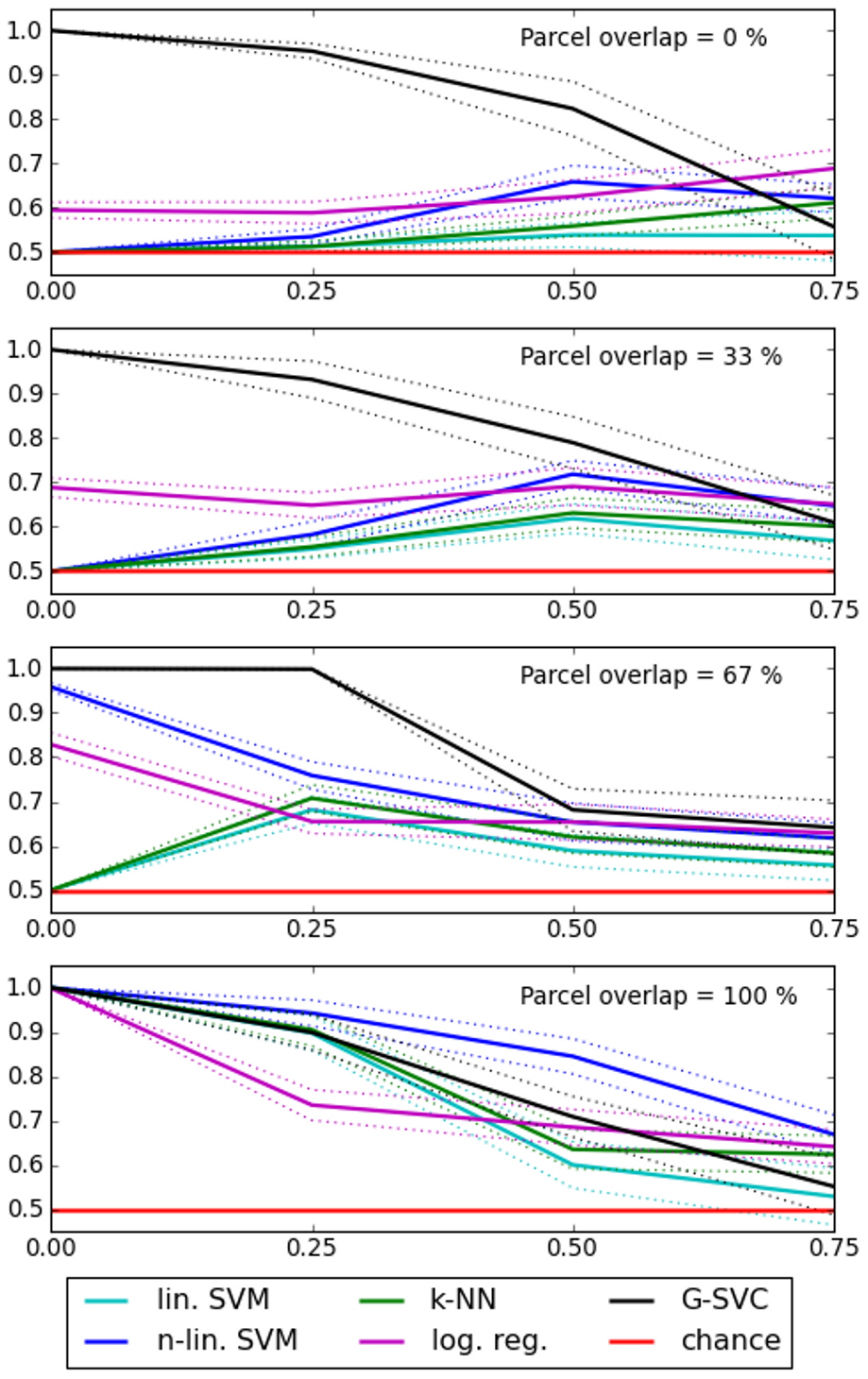
Artificial data: influence of the nature and amount of inter-subject variability on the mean accuracy (± standard error). Chance level is 0.5. From top to bottom, the variability in geometrical atributes (parcel location, which controls the parcel overlap) decreases. On the x axis of each subplot, the variability in activation levels increases from left to right. G-SVC is the only method that can handle geometrical variability (the four black curves are almost identical).

In the artificial datasets, the true number of parcels composing the patterns is known by construction (three). Therefore we used the corresponding number of nodes, *q* = 3, in the graph construction phase of our G-SVC framework. We compared the results given by G-SVC with the performances of the different vector-based benchmark methods. For each *variability case*, we used *test1* to assess whether G-SVC performs at a different level than each of the vector-based benchmark methods. The mean results (across the twenty datasets randomly generated for each *variability case*) are presented in Fig. 6.

When no geometrical variability is present (100% overlap of the middle parcels, lower plot on Fig. 6), all methods performed similarly. In these cases, the accuracy decreases when the variability in the activation levels increases, for all methods. This can be explained by the fact that when 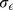 increases, the discriminability of the patterns decreases; it is even possible that, depending on the sample values drawn for 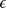, the characteristic contrast (the fact that the middle parcel is more activated when *y* = 1 than when *y* = 2) becomes inverted in one subject compared to the other one; in this case, patterns from the two conditions in a given subject are not discriminable from what was learnt in the other subject.

When the level of geometrical variability increases (i.e. when the percentage of overlap of the middle parcels decreases), the accuracy levels of the vector-based method decreases, whereas the performance of G-SVC is not affected. Indeed, statistical differences (*test1*, *p*<0.05) between G-SVC and all vector based methods are observed for 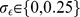 when the overlap is lower than 100%; for 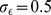, G-SVC also outperforms all methods for a 0% overlap, and some of the vector based methods for intermediate overlap levels (33% and 67%).

Note that we also conducted experiments with *q*>3 for G-SVC, which in some cases produced slightly higher accuracy levels. But the mean differences with the results obtained with *q* = 3 (black curves of Fig. 6) were not significant; the equivalent curves were not distinguishable from the black ones, and are therefore not displayed.

Overall, we can conclude that G-SVC is the only method that deals with geometrical variability in the functional features of the patterns (i.e. in this case, when the middle parcels do not fully overlap), and that it handles variability in the activation levels as well as the vector-based methods.

### 3.2 Results on real data - G-SVC vs. vector-based methods

Since in this case, we do not know the exact number of underlying parcels to be used to model the patterns, we ran G-SVC with a fixed number of nodes *q* for all subjects, and repeated the analysis for 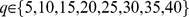. We chose the smaller value of *q* to be five because according to the functional architecture of the primary auditory cortex, the five stimuli used in our experiment should result in at least five different activated regions [68], [69]. Table 1 contains the performances of G-SVC vs. the vector-based benchmark methods. For the benchmark methods, the reported score is the highest accuracy obtained across all values of their hyper-parameters; for G-SVC, we report the highest and lowest accuracy levels obtained across all values of *q*. All vector-based methods performed fairly similarly, with accuracy levels between 0.27 and 0.31, i.e. slightly higher than chance level (0.2). G-SVC yielded higher level of accuracies in all cases, with performances between 0.39 and 0.56, depending on *q*. In the right hemisphere, the performance differences between both the highest and lowest accuracies obtained with G-SVC and any benchmark methods is statistically significant (*test1*, *p*<0.05). In the left hemisphere, the highest and lowest accuracies of G-SVC are significantly higher (*test1*, *p*<0.05) than the best accuracies produced by linear SVM, and the l1- and l2-logistic regressions; the mean differences between the accuracy of G-SVC and the ones of nonlinear SVM and k-nearest neighbors are large (the lowest G-SVC score is 0.39, compared to 0.30 for nonlinear SVM and k-NN), but not significant.

**Table 1:** Real data: mean inter subject prediction accuracy of G-SVC (highest/lowest, across *q*) vs. benchmark vector-based methods (best case).

	G-SVC	lin. SVC	n-lin. SVC	k-NN	log. reg.
right HG	**0.56/0.44**	0.31	0.30	0.28	0.28
left HG	**0.47/0.39**	0.27	0.30	0.30	0.28

Chance level is 0.2.

### 3.3 Results on real data - G-SVC vs parcel-based methods

**Figure 7:**
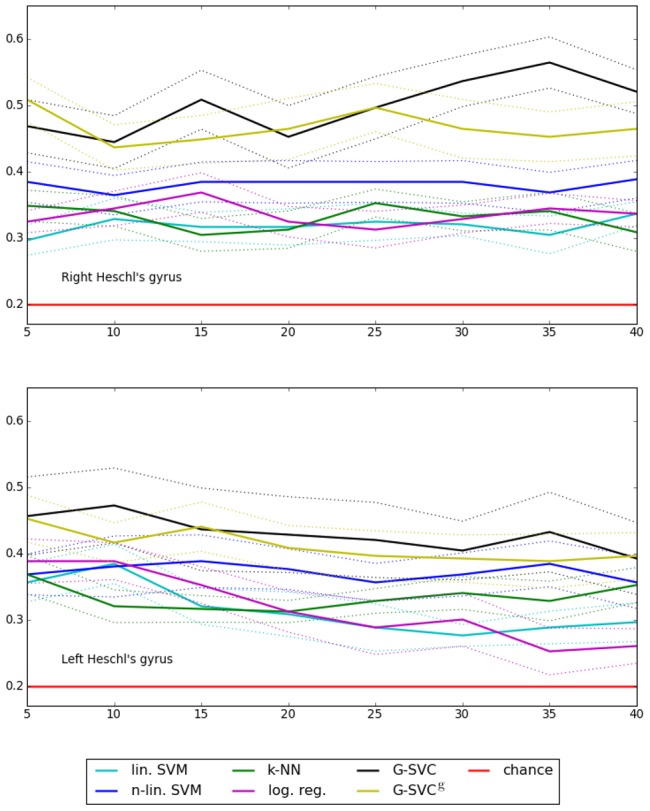
Real data: mean accuracy levels (± standard error) as a function of the number of parcels *q*, for G-SVC, G-SVC^g^ and other parcel-based approaches. Chance level is 0.2. G-SVC and G-SVC^g^ clearly outperform the other methods, and display good robustness to the choice of the number of graph nodes.

In this experiment, we compared the results given by G-SVC, for which an individual parcellation is computed on each subject to estimate our graphical representations, to the ones obtained with methods where the parcellation is identical in all subjects (G-SVC^g^ when using graphical representations computed from the group-parcellation, and other parcel-based benchmark methods). All methods share a common parameter, the number *q* of parcels. Fig. 7 shows the accuracy curves obtained with the different methods in fonction of *q* (the maximum and minimum values of the black G-SVC curves correspond to the values reported in Tab. 1). We used *test2* to assess whether the accuracy curve of G-SVC is different from the ones given by the benchmark parcel-based methods.

As clearly visible in Fig. 7, the accuracy curves of both G-SVC and G-SVC^g^ are above the ones given by all other methods; using G-SVC as reference, this difference is significant for all methods in the right hemisphere (*p*<0.05); in the left hemisphere, the difference with the accuracy of the logistic regression is significant (*p*<0.05), shows a non-significant trend with linear SVM (*p* = 0.07, but is not significant with the other methods. These results clearly demonstrate the added-value of using our graphical representations associated with our graph kernel to handle inter-subject variability. Furthermore, the accuracy curves of G-SVC are slightly above the ones of G-SVC^g^. Even though this difference is not statistically significant, this trend might suggest that using individual parcellations to construct our graphical representations yields more accurate representations of the underlying activation patterns.

### 3.4 Results on real data - G-SVC with variable number of nodes

We performed another set of experiments in order to test the ability of our G-SVC framework to learn from a set of graphs having different number of nodes. For each experiment, we randomly draw the number of nodes for each subject, in {5,10,15,20,25,30}. Therefore, in each fold of the cross-validation, the training set contained graphs with different number of nodes, and the generalization power was measured on graphs from the left-out subject, i.e. with yet a different number of nodes. Twenty such experiments were conducted. The average accuracies over these twenty experiments were 0.47±0.02 for the right Heschl's gyrus, and 0.42±0.02 for the left Heschl's gyrus. These numbers are between the highest and lowest accuracies obtained with fixed *q* for all subjects (see Tab. 1). In only 6 of the 40 cases (20 for each hemispheres) was the accuracy significantely lower than the highest one obtained with fixed *q* (*test1*, *p*<0.05). This set of experiment shows that G-SVC can handle graphs with different number of nodes, and therefore is robust to some structural variation between the graphical representations learnt from different observations/subjects.

### 3.5 Results on real data - Influence of each graph attribute

**Figure 8:**
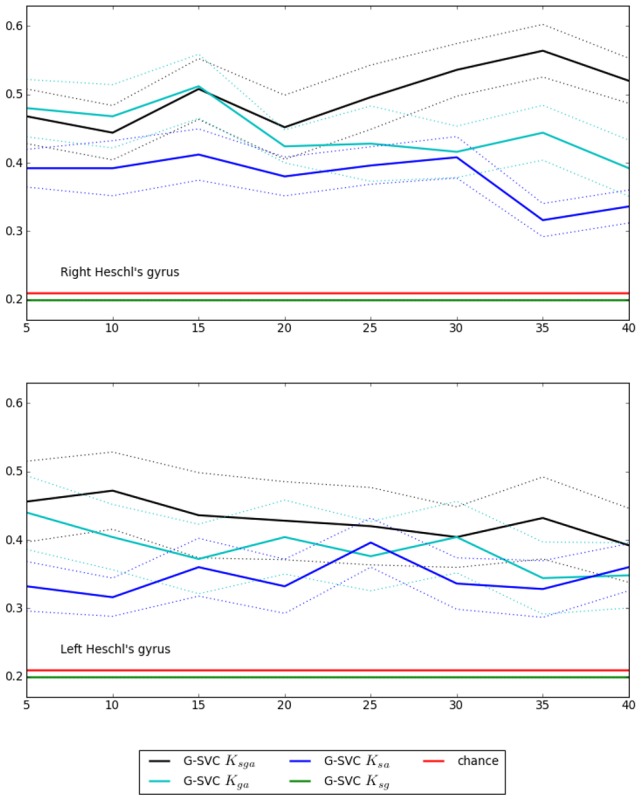
Real data: influence of the nature of the exploited graph characteristics. Mean accuracy (± standard error) of G-SVC as a function of the number of nodes *q*. Chance level is 0.2. The best results are obtained when the three types of information are exploited, i.e. with the *K_sga_* kernel.

The graphical representations 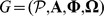 used in our G-SVC framework comprise information about the activation levels, the location and the spatial structure of the functional features extracted from the patterns, respectively carried by the nodes attributes 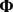 and 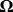, and the adjacency matrix **A**. Here we want to study whether these three types of features are necessary to achieve accurate classification. Similarly to the definition of our full kernel *K_sga_* given in Eq. (6), one can define three kernels *K_sg_*, *K_sa_* and *K_ga_* for which one of the three types of graph attributes is ignored by removing the corresponding base kernel from Eq. (6). For instance, 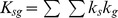. Fig. 8 shows the global classification accurary of G-SVC when using all features (i.e. using kernel *K_sga_*) vs when using two out of the three types of features (i.e. using kernel *K_sg_*, *K_sa_* or *K_ga_*). If one does not use the activation attributes (i.e. when using *K_sg_*), keeping only the geometric and structural features, the performances are systematically at chance level, showing that, as expected, the activation attributes are necessary to achieve classification. If one does not use geometric or structural information (i.e. by using *K_sa_* or *K_ga_*), the mean performance curve is lower than when using all three types of information; this difference is statistically significant (*test2*, *p*<0.05) in the right Heschl's gyrus, but not in the left one.

The construction of our *K_sga_* kernel, described in section 2.3, explains the intuition behind the use of each of the three types of information. Here we have demonstrated experimentally that indeed, if any of the three base kernel is removed, the performances decrease. This shows that our full kernel *K_sga_* indeed provides the best generalization results, which means that it exploits the information contained in all of the activation, geometric and structural attributes. This result also confirms that our main assumption, namely that the spatial organization of the activation patterns is consistent across subjects, is indeed true, and that our kernel allows exploiting this property efficiently to perform inter-subject predictions.

### 3.6 Results on real data - Kernel parameters

The kernel that we designed includes two hyper-parameters, the bandwidths 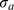 and 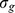. We adapted a standard heuristic to estimate their value on the training set, which was used on all experiments. This process implies having different values for 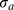 and 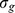 in each fold of the cross validation. We therefore computed the mean and standard deviation of the estimated bandwidths across folds of the cross validation, for all experiments performed on the real data with fixed *q* for all subjects. This yielded 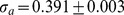 and 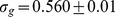 for the analyses performed in the right hemisphere, and 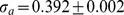 and 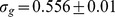 in the left Heschl's gyrus. We observe that on the one hand, the estimated bandwidth values are very stable across folds, and on the other hand, they are almost identical in the right vs. the left hemisphere. These results combined with the overall positive results given by G-SVC validates the effectiveness of the heuristic that we proposed to choose the values of the kernel parameters.

## Discussion

In this paper, we have demonstrated that our G-SVC framework can be used to learn an accurate predictor to perform inter-subject classification of fMRI activation patterns. Experiments conducted on artificial datasets (presented in 3.1) showed that G-SVC is the only method that deals with varying locations of the functional features of interest across subjects. Experiments on a real dataset suffering from large inter-individual functional variability showed that G-SVC performed better that all tested vector- and parcel-based methods (respectively in 3.2 and 3.3); in particular, the latter proved the added-value of a graph-based framework based on individual representations compared to using a common parcellation for all subjects. We also showed that G-SVC is robust to changes in the number of graph nodes *q*, both if *q* is identical for all subjects (in 3.3) or not (in 3.4), the latter demonstrating the robustness of our kernel to some potential structural differences. Furthermore, we showed in 3.5 that G-SVC performs best when it uses all the information available in the graph (i.e. the activation, geometric and structural characteristics of the input functional patterns), which demonstrates the soundness of our learning scheme to produce group-invariant graphical representations and the ability of our graph kernel to exploit the consistency of the spatial organization of the activation patterns across subjects when this assumption is verified.

### 4.1 Hyper-parameters estimation

The G-SVC framework comprises three hyper-parameters: the bandwiths 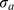 and 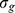 of the functional and geometric base kernels used to design the full *K_sga_* kernel, and the number of nodes *q* of the constructed graphs. For the former two, we have proposed a heuristic that estimates the values of 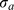 and 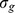 on the training dataset. This heuristic selects the median distance between the corresponding attribute values of the observations of the training set. It is simple to implement and the results shown in this paper demonstrate its effectiveness, thus providing an easy way to automatically select the values of these two hyper-parameters.

Regarding the number of graph nodes *q*, we hereafter describe three potential strategies for choosing *q* that explore three different directions. First, we have shown that when *q* is chosen identical for all subjects, G-SVC is robust with regards to the selected value since it significantely outperforms benchmark methods for almost all values of *q* (see Fig. 7). In order to choose the value of *q*, one can therefore exploit prior knowledge about the functional properties of the studied region, as we did in this study with the architecture of the primary auditory cortex [68], [69]. Second, in paragraph 0, we also showed that G-SVC can handle input graphs with different number of nodes *q*. This suggests that one could attempt to work at the individual level to optimize the graphical representation of such distributed patterns. One simple strategy to do so could be to apply a standard univariate analysis on each subject of the training set, and use the number of significantly activated clusters across all experimental conditions as a lower bound for *q*. Finally, if one need a fully automatic strategy, the value of *q* can be chosen in a nested cross validation amongst a given list of values that can be constructed using any of the aforementioned strategies.

### 4.2 Linear vs nonlinear classifiers

Since G-SVC uses a graph kernel to find a nonlinear decision boundary in the original data space, it is in fact a nonlinear SVM classifier. The usefulness of such nonlinear classifiers for neuroimaging applications is the subject of an on-going debate in the litterature (see the introduction of [79] for a summary). The appeal of linear classifiers for fMRI applications is mainly twofold: i) they facilitate the interpretation of the classification results, for instance thanks to the ability to directly visualize weight maps when working with linear SVM [80]; and ii) despite their simplicity, their performances always are amongst the highest [81]. Regarding the first point, [79] offers a solution to ease the interpretation of the results given by nonlinear classifiers by visualizing sensitivity maps. As for the second point, our study is a new example where a nonlinear method clearly outperforms linear classifiers.

While it is not the focus of this paper, note that our framework is directly applicable to the equivalent within-subject learning task (see results in Table S1). In this easier task, G-SVC produces mean accuracy levels that are not statistically higher than those of vector-based methods.

Therefore G-SVC allows reaching higher accuracy rates than benchmark methods for the inter-subject classification task, but not for the within-subject one. We believe that it is the case because i) we have identified a factor that contributes to the poor performances of standard, and in particular linear, methods (the inter-individual variability) for the selected learning problem (inter-subject classification), ii) we use prior knowledge to model the influence of this factor and exploit it into the design of a nonlinear classifier (here, to construct a graph kernel), and iii) we allow the classifier to work in a fairly low-dimensional space (our graph construction scheme uses a small number of parcels, and therefore acts as a dimensionality reduction process), thus offering a reasonable ratio between the sample size (number of observations available) and the dimensionality of the space in which the classification is performed. These three points could constitute a set of rules that can help identify questions for which it might be worth developing nonlinear classifiers in neuroimaging.

Moreover, this interpretation is consistent with another explanation for the potential usefulness of nonlinear methods, that was for instance described in [82]. Building upon the example of the quadratic kernel, which is equivalent to adding features equal to the products (i.e. the *interactions*) between the original features, one could understand the added value of nonlinear models when such interactions are related to the experimental variable *y* to be predicted. In the case of our G-SVC framework, the graphical representation explicitely encodes some these potential interactions by linking spatially adjacent parcels: therefore, instead of using all interactions terms between original features (as with the quadratic kernel), only a subpart of these interactions are considered, those for which there exist an edge between graph nodes. Our G-SVC framework takes advantage of these interactions because the *K_sga_* kernel directly uses the information carried by the graph edges.

### 4.3 Examining assumptions and potential applications

The G-SVC framework in its most generic form comprises an unsupervised learning step that constructs attributed graphs built upon a parcellation and a supervised learning step using a carefully designed kernel. It is therefore applicable as soon as it is possible to learn a meaningful parcellation for the problem of interest (classification, regression, clustering etc.). If one wants to extend the framework to other applications than the one described in the present paper, one simply needs to determine what is the information of interest in the parcels (in order to define the attributes of the graph nodes), what criterion to choose to build the graph structure (spatial adjacency, connectivity) and then to define one base kernel for each type of graph attributes.

As implemented in the present paper, our G-SVC framework relies on the main assumption that the spatial organization of the activation patterns is consistent across subjects. Indeed, our model of functional variability lets the intensity levels and locations of functional features vary across subjects, but their relative positions is supposed to be invariant across subjects, which we enforce by looking for region adjacency graphs that have a common structure. The question is then to know under what circumstances this assumption holds true. At the full brain scale, it is well known that the macroscopic organization of the cerebral cortex is reproducible across subjects, as for instance demonstrated by the reproductibility of the respective positions of the Brodmann areas, together with their functional specificity. We therefore believe that our G-SVC framework should be directly applicable to study large scale activation patterns based on full brain individual parcellations such as provided in *freesurfer* [73].

Studying neural representations at a finer scale is a crucial issue in modern neuroscience [2], [25], [44], [83]. The topographical organization of primary sensory areas (see for instance [68], [69] for the auditory cortex) ensures the consistency of the spatial organization of activation patterns across subjects. The successful results provided by our framework show that G-SVC is able to overcome the large functional variability encountered at such fine spatial scale; furthermore, it constitutes yet another confirmation that fMRI is able to capture the spatial organization of the auditory cortex and it shows that its topography is indeed reproducible across subjects. Furthermore, it is to be noted that G-SVC yields higher classification performances in the right vs. the left Heschl's gyrus. This might reflect a lateralization in the functional specialization of the auditory cortex, such as described in [84], but such a claim would need further investigation.

In general, G-SVC is therefore a tool perfectly suited to study the consistency of representations in all sensory areas, and also the influence of a pathology on the organization of processing in these areas (see an example with macular degeneration in [85]). The question whether our main assumption is still valid in higher level cortical areas remains open, and the methods that attempt to deal with functional variability at such fine spatial scale take different routes with respect to this question. The so-called hyper-alignement (hereafter HA, [25]), maps the activation patterns of different individuals to a common “high dimensional” space *without* enforcing the preservation of the spatial organization of the input patterns. Even if HA has proved successful in inter-subject decoding tasks, its success does not demonstrate per se the existence of idiosyncrasies, i.e. subject-specific architectures of the activation patterns at the scale offered by fMRI voxels. Indeed, finding common representations across subjects is a learning task that is simply easier to solve when relaxing the constraint on the spatial organization as in HA. Another method, the function-based alignment (hereafter FBA, [23]) estimates an explicit set of anatomical correspondences between brain voxels of different individuals by matching full-brain functional patterns. The use of a diffeomorphic model to estimate this spatial transformation implicitly requires the spatial organization of activation patterns to be consistent across subjects *over the whole cortex*; the positive results obtained by FBA would tend to validate this assumption, but it remains to be seen whether such method allows to improve prediction accuracy in inter-subject MVPA.

The successful results of the G-SVC framework described in the present paper indicate that it is possible to learn representations that have a common spatial organization across subjects and use them to perform inter-subject MVPA. An interesting feature of our framework is that it bypasses the need of explicit correspondence between brain voxels, which are hard to establish due to the conjunction of shape variability and variable functional organization of anatomical areas. When the delineation of a cortical area is so variable across individuals that it requires the use of functional paradigms known as *localizers* (see examples in [86]), our G-SVC framework will allow studying fine scales representations within these functionally defined regions, thanks to its ability to work with regions that are not matched across subjects. Finally, contrarily to FBA and HA that estimate their respective models on a dedicated experiment during which the subjects watched a movie, G-SVC does not require such dedicated “calibration” experiment to perform inter-subject predictions. These three methods are therefore somehow complementary, and we believe that comparing their behaviors and results should prove useful to assess the consistency of the spatial organization of brain patterns across individuals, but it is clearly beyond the scope of this paper.

### 4.4 Which graph kernel for fMRI graphs?

In the last two years, the use of graph kernels has emerged as a new tool to handle graphical representations estimated from fMRI data. We here try to summarize the different routes taken in the few published studies and provide directions that could help shape future work. We start by making a clear distinction between the different nature of the input data, namely whether the graphs have been constructed from connectivity (resting state fMRI) or activation (task-based fMRI) studies.

Indeed, connectivity graphs are most often constructed by thresholding a correlation (or other similar criteria) matrix [37], which makes them inherently unlabeled. In this context, one can expect that it is the topological properties of the graphs that carry most of the predictive power for the learning task at hand. This is the case when trying to characterize populations of patients for which the pathology has affected the connectivity of the brain, which is a major issue in clinical neuroscience. Most existing graph kernels make use of such properties, and when the effect of the pathology is global, one can therefore rely on the vast graph kernels literature that deals with unlabeled graphs. Note that the unifying framework described in [87] has allowed to demonstrate that a large number of previously designed graph kernels are actually instances of the *R*-convolution kernel family that we instanciated in the present paper. Furthermore, those kernels are often tunable to handle labeled nodes. If one need to add local information to better handle pathologies which result in focal, rather than global, connectivity dysruptions, an easy strategy consists in adding labels on the nodes, as done in [38]. In such case, the efficiency of the Weisfeiler Lehman kernel has made it popular in the emerging litterature of graph kernels applications for fMRI data [38], [39], [41], [42].

In contrast, when working with task-based fMRI data, the predictive power is conveyed by the amplitude differences of the BOLD signal. The most natural way to encode this information in graphical representations is to derive activation features from the BOLD signal (for instance contrast maps estimated with a univariate GLM) and use them as real-valued attributes of the graph nodes. In that case, because the activation differences of interest are often very small, it is crucial to avoid any quantization or discretization of these nodes attributes. It therefore becomes necessary to use graph kernels that handle real-valued attributes, for which the litterature is somewhat smaller. The most popular kernel in this categorty is the shortest path kernel [88], which was successfully used in [40]. In the present study, we took another route by constructing a dedicated kernel as yet another instance of the *R*-convolution kernel family, and demonstrated that such approach can yield accurate inter-subject predictions.

In all cases, fMRI graphs usually have a relatively small number of nodes (at most a few hundreds for graphs generated from full brain parcellations) compared to the more classical applications of graph kernels (world wide web networks, chemo- and bio-informatics) for which graphs often have thousands and sometimes millions of nodes. Even if this allowed us to focus our design scheme on the expressivity of the kernel, rather than its computational efficiency, it remains crucial to use kernels that scale up efficiently to a few hundreds of nodes. The complexity of the classical shortest path kernel is in *O*(*q* ^4^), where *q* is the number of graph nodes. Our kernel scales up as *O*(*q^2n^*), where *n* is the size of the considered subgraphs, which gives *O*(*q* ^4^) for the implementation given in the present study (with edges as subgraphs, i.e for *n* = 2). Since all kernels scale up linearly with the number of examples, using such kernels in *O*(*q* ^4^) might require several (dozens of) hours, depending on the number of examples. Therefore it remains important to improve the efficiency of the kernel computation. One could envision using recently developed kernels that deal with real-valued attributes and that are significantely more efficient than *O*(*q* ^4^) [89], [90]. In the case of our intuitively designed *K_sga_* kernel, which compares all of the chosen types of subgraphs to all other subgraphs, one way to gain in efficiency would be to use the location of the nodes to limit the number of comparisons (i.e by comparing a given subgraph only to the ones located close-by). This should also improve its expressivity by avoiding meaningless comparisons of edges that are far away from each other. This strategy could also make it possible to use more complex subgraphs (triplets etc.) while maintaining affordable run times, although it remains to be investigated whether it would improve the prediction accuracy.

## Conclusions

We described a new graph-based structured learning scheme designed to overcome inter-individual variability present in functional MRI data. Our approach constructs attributed graphs to represent distributed functional patterns and performs inter-subject classification to predict an experimental variable from the data. The graph construction scheme that we introduced starts with a parcellation, and then encodes the relevant characteristics of the parcels (their locations, activation levels and spatial organization) into the graph. The classification is performed with support vector machines using a custom-designed kernel that exploits all the attributes of the graphs. Results on artificial datasets generated to parametrically control the amount of inter-individual variability along two dimensions (the location and intensity of functional features) showed that our G-SVC framework is the only method able to yield satisfactory performances when the locations of functional features vary across subjects. Results on real data showed that G-SVC outperforms both vector- and parcel-based state of the art classification methods, that it is robust to the number of graph nodes in the observations, both if it is chosen constant for all observations in the different subjects, or if it is different across subjects. As implemented in this paper for fMRI data, this framework is a tool of choice to study local neural representations at fine spatial scales in regions that are not well aligned across individuals, which is a crucial problem in modern neuroscience. Moreover, it is easily adaptable to other types of learning problems posed by different neuroimaging modalities.

## Supporting Information

Table S1 **Real data: within subject mean accuracy of G-SVC (highest/lowest across** ***q*** **) vs. benchmark vector-based methods (best case).** Chance level is 0.2.
(PDF)
