# Supplementary material for: Graph-Based Inter-Subject Pattern Analysis of fMRI Data
Source: PLoS One. 2014 Aug 15;9(8):e104586. doi: 10.1371/journal.pone.0104586 (PMC4134217; doi:10.1371/journal.pone.0104586)
Supplement: Table S1 — Real data: within subject mean accuracy of G-SVC (highest/lowest across q ) vs. benchmark vector-based methods (best case). Chance level is 0.2. (PDF) [file pone.0104586.s001.pdf]

Supplementary Information: Within-subject classification results

Even though our G-SVC framework is designed to deal with inter-individual variability, it is directly usable in a within-subject analysis. We here present the results of G-SVC in the within-subject classification task, i.e to predict the class of stimulus that was presented to the subject for a given fMRI pattern. We used the same graph representations as for the inter-subject learning task and repeated the analysis for different number of nodes $q \in \{5, 10, 15, 20, 25, 30, 35, 40\}$. We used a leave-one-session-out cross-validation scheme and report the average global classification accuracy obtained across folds. The results are reported in Table S1, and compared to results given by the vector-based benchmark methods. The accuracy levels obtained with G-SVC are not statistically different from the performances given by standard vector-based methods. Overall, although the maximum (across values of q) mean accuracy obtained with G-SVC is higher than the performances given by any benchmark methods in both hemispheres, these differences are not statistically significant.

	G-SVC	lin. SVC	n-lin. SVC	k-NN	log. reg.
right HG	0.56 / 0.52	0.51	0.46	0.42	0.50
left HG	0.57 / 0.51	0.48	0.42	0.41	0.51

Table S 1: Real data: within subject mean accuracy of G-SVC (highest / lowest across q) vs. benchmark vector-based methods (best case). Chance level is 0.2.
